# Impact of Drying Process on the Phenolic Profile and Antioxidant Capacity of Raw and Boiled Leaves and Inflorescences of *Chenopodium berlandieri* ssp. *berlandieri*

**DOI:** 10.3390/molecules28207235

**Published:** 2023-10-23

**Authors:** Ángel Félix Vargas-Madriz, Aarón Kuri-García, Ivan Luzardo-Ocampo, Haidel Vargas-Madriz, Iza Fernanda Pérez-Ramírez, Miriam Aracely Anaya-Loyola, Roberto Augusto Ferriz-Martínez, Octavio Roldán-Padrón, Luis Hernández-Sandoval, Salvador Horacio Guzmán-Maldonado, Jorge Luis Chávez-Servín

**Affiliations:** 1 Laboratorio de Biología Celular y Molecular, Facultad de Ciencias Naturales, Universidad Autónoma de Querétaro, Av. De las Ciencias S/N, Juriquilla, Querétaro 76230, Mexico; angel.vargas@uaq.mx (Á.F.V.-M.); aaron.kuri@uaq.mx (A.K.-G.); roberto.augusto.ferriz@uaq.mx (R.A.F.-M.);; 2 Tecnológico de Monterrey, The Institute for Obesity Research, Av. Eugenio Garza Sada 2501, N.L., Monterrey 64841, Mexico; ivan.8907@gmail.com; 3 Tecnológico de Monterrey, School of Engineering and Sciences, Av. General Ramón Corona 2514 Nuevo Mexico, Zapopan 45138, Mexico; 4 Departamento de Producción Agrícola, Centro Universitario de la Costa Sur, Universidad de Guadalajara-UDG, Av. Independencia Nacional 151, Autlán, Guadalajara 48900, Mexico; 5 Facultad de Química, Universidad Autónoma de Querétaro, Cerro de las Campanas S/N, Querétaro 76010, Mexico; 6 Laboratorio de Nutrición Humana, Facultad de Ciencias Naturales, Universidad Autónoma de Querétaro, Av. De las Ciencias S/N, Juriquilla, Querétaro 76230, Mexico; 7 Laboratorio de Botánica, Facultad de Ciencias Naturales, Universidad Autónoma de Querétaro, Av. De las Ciencias S/N, Juriquilla, Querétaro 76230, Mexico; luishs@uaq.mx; 8 Laboratorio de Alimentos, Centro de Investigación Regional del Centro, INIFAP, Campo Experimental Bajío, Km 6, Carr. Celaya-San Miguel de Allende, Apdo. Postal 112, Celaya 38110, Mexico

**Keywords:** *Chenopodium berlandieri* ssp. *Berlandi*, lyophilization, proximal chemical analysis, oven-drying, phenolic compounds, quelites

## Abstract

*C. berlandieri* ssp. *berlandieri* (*C. berlandieri*) is one of the most common members of the group of plants known as quelites, which are dark leafy greens widely consumed in Mexico. This study aimed to evaluate the impact of two drying procedures (oven drying and freeze-drying/lyophilization) on the polyphenolic composition, antioxidant capacity, and proximal chemical analysis of *C. berlandieri* leaves and inflorescences (raw or boiled). The results indicated that the raw freeze-dried samples had higher amounts (*p* < 0.05) of total phenolic compounds, total flavonoids, and antioxidant capacity, mainly in the inflorescence. The oven-dried samples showed an increased concentration of polyphenols after boiling, while the lyophilized samples showed a slightly decreased concentration. The drying process was observed to have little impact on the proximal chemical composition. Quantification by UPLC-DAD-ESI-QToF/MS identified up to 23 individual phenolic compounds, with freeze-dried samples showing higher amounts of individual compounds compared with oven-dried. Procyanidin B2 was found exclusively in the inflorescences. The inflorescences have a higher content of phenolic compounds and greater antioxidant capacity than the leaves. Regardless of the drying process, the leaves and inflorescences of *C. berlandieri* contain an interesting variety of phenolic compounds that may have beneficial effects on health.

## 1. Introduction

Humankind has relied on plants as a food source since its earliest origins. Modern science has set itself the task of analyzing plants’ nutritional composition and identifying their bioactive compounds. Compounds of interest are phenolic compounds, considered natural antioxidants with the potential to prevent the risk of non-communicable diseases such as cancer, type 2 diabetes, and cardiovascular diseases, among other conditions [1]. By identifying the bioactive compounds of edible plants, scientists can improve dietary recommendations and identify the plants that are the best sources of these components. One such plant is the quelites group, comprising more than 500 native Mexican plants whose leaves, tender stalks, and inflorescences have been used as a food source in Mesoamerica since pre-Hispanic times. These include *Amaranthus* spp., (amaranth), *Portulaca oleracea* (purslane, known in Mexico as verdolaga), *Cnidoscolus aconitifolius* (tree spinach, or chaya), *Chenopodium berlandieri* ssp. *nuttaliiae* (huauzontle), and *Chenopodium berlandieri* ssp. *Berlandieri* (quelite cenizo) [2,3].

The last of these, quelite cenizo, is one of the most widely consumed plants in Mexico, and it is used extensively in several typical dishes and preparations [4]. Moreover, its leaves and inflorescences presumably exhibit antibacterial, chemopreventive, hepatoprotective, anti-inflammatory, and antioxidant effects, based on analyses of other Chenopodium species [2]. The leaves and inflorescences can be consumed raw, as decoctions (boiling process), fried or dried, or as spices to improve the flavor, color, or texture characteristics of the food preparations in which they are used [5].

It is well known that the biological effects of phenolic compounds depend largely on their chemical stability and the manner in which the plant that contains them is processed. Environmental effects, such as ultraviolet radiation, temperature, and oxygen, must be considered, as well as the intrinsic characteristics of the product, including water activity or processing conditions, such as the type of drying and heat treatments used in culinary or industrial processes [6]. Drying a plant sample lengthens its shelf life and can preserve the quality and quantity of the components of the sample, and in the food industry, drying is an essential method for inhibiting microbial growth [7]. However, some types of drying can affect nutritional compounds due to the enzymatic and non-enzymatic effects that occur during the drying process [8]. Prevalent drying methods include convection drying with hot air and freeze-drying [7]. Various studies have shown that hot-air drying has a negative effect on nutritional components because it degrades the proteins found in plant samples. Freeze-drying, in contrast, removes moisture from samples through sublimation, but it is an expensive process due to its high energy consumption and in some samples it has been observed that the freezing process can modify the properties of fiber in foods [9]. Most quelites can be dried to extend their shelf life by reducing enzymatic and non-enzymatic processes. Different drying methods may be used: sun drying, oven drying, and lyophilization. As it is known that ultraviolet radiation can degrade phenolic compounds and other components with antioxidant capacity, this method is not currently recommended, but it is useful to compare how the content of phenolic compounds is affected by two other methods: oven drying and freeze drying. Likewise, it is important to document how the culinary heat treatment used in the preparation of the plant influences the content of phenolic compounds in edible botanical structures, such as leaves and inflorescence [8]. Finally, in *C. berlandieri*, the leaves are consumed more often than the inflorescences, so it would be useful to determine which botanical structure holds the greater quantity of phenolic compounds. This research aimed to assess the impact of two drying methods (oven drying and lyophilization) on the proximal chemical analysis, phenolic profile, and antioxidant capacity of raw and boiled leaves and inflorescences of *C. berlandieri*.

## 2. Results and Discussion

### 2.1. Impact of Drying Method on the Proximal Composition of C. berlandieri Leaves and Inflorescences

Table 1 shows the proximal chemical composition of the leaves and inflorescences of *C. berlandieri* processed by oven drying and lyophilization. No significant differences (*p* > 0.05) were found in the moisture or protein content of the samples analyzed. The leaves had higher ash content (*p* < 0.05) than the inflorescences, but inflorescences contained a higher amount of total carbohydrates (*p* < 0.05).

There was no clear trend in the impact of the drying process on chemical composition, but both types of lyophilized inflorescences (BLI and RLI) displayed higher amounts of lipids and dietary fiber than the other groups. The protein values obtained are higher than those indicated by Santiago-Saenz et al. [10] in samples of the same species (3.45%), and even in other species such as *Amaranthus hybridus* (1.81%) and *Portulaca oleracea* (3.65%), but it should be noted that the values of that study are reported for fresh matter. Considering the protein values reported for the leaves of the *quelite cenizo* (26.2%) [11], protein loss could be attributed to the drying process [12]. Drying method can positively or negatively affect the nutritional value of foods; in most cases, freeze- drying is better than oven drying for ensuring that plant samples retain their content of nutrients and bioactive compounds [13]. Although the scientific literature mentions that freeze-drying is a gentle dehydration method, it has been observed that, in some foods, it can cause certain changes in their physicochemical properties, even more than oven drying [14]. In a study carried out by Li et al. [15], freeze-drying of Vocia faba Linn (beans) removed more protein content than oven-drying, although the authors noted that the result may have been influenced by the drying time as well as the pre-freezing of the sample at a temperature of −80 °C. In a study by Oliveira-Alves et al. [12], in contrast, a decrease in the amount of protein was observed after oven drying samples of Salicornia ramosissima (sea asparagus); the authors mention a possible denaturation of the protein at a temperature of 70 °C for 72 h. However, other studies with different plant samples—including the present study—do not report significant differences [16].

Still, even when dried, *C. berlandieri* leaves and inflorescences show higher protein content than conventional edible leaves, such as cabbage (12.8%) and lettuce (14%) [17]. Differences in dietary fiber content could be attributed to chemical changes linked to polysaccharide reordering, which would increase the dietary fiber content of raw oven-dried leaves (ROL). However, the amount found in raw lyophilized inflorescences (RLI) could be comparable to the fiber content found in lyophilized and dried cauliflower (20.6 and 19.1–19.8%, respectively) [18], where the authors found that the degree of esterification of pectic polysaccharides experienced a significant heat-induced dietary fiber change. Dietary fiber is composed of water-soluble polymers (pectins, gums) and insoluble polymers (cellulose, hemicellulose, lignin) [19]. Some studies have found that heating foods can cause a breakdown of the cell matrix of the fiber plant sample [7]. Heat drying can cause the hydrolyzation of structural pectin or protopectin, transforming them into soluble pectins, thus increasing the amount of soluble fiber. However, this type of drying can also cause the degradation of hemicellulose and cellulose, transforming them into simple carbohydrates by decreasing the amount of insoluble fiber [20]. Freeze-drying can modify some fiber components (insoluble pectins) by activating the enzyme pectin-methyl-esterase, causing de-esterification of the fiber components and generating digestible carbohydrates [9]. It has been observed that the type of drying, whether hot or cold, can increase or decrease the amount of fiber in different plant samples [19], as observed in the present study. This is particularly true, for the purposes of our study, for ash content, where obtained values are higher than other quelites [10,21], even when dried [17].

### 2.2. Impact of the Drying Process on the Phytochemical Composition and Antioxidant Capacity of Raw and Boiled Leaves and Inflorescences of C. berlandieri

Inflorescences showed the highest total phenolic compounds (TPC), total flavonoids (TF), and the highest antioxidant capacity quantified by the DPPH, ABTS, and FRAP (Table 2) methods.

As observed, boiling reduced TPC and FT, but resulted in higher FRAP in freeze-dried leaves than other treatments. In both types of plant material, freeze-drying contributed to preserving TPC and TF as well as antioxidant capacity. It has been reported that freeze-drying preserves the polyphenolic composition of food matrices and prevents their oxidation due to the combined effect of vacuum and low temperature [22]. There are no studies on the impact of drying methods on the phytochemical composition of the leaves and inflorescences of *C. berlandieri*. The TPC and TF contents obtained in this study are lower than those reported for the leaves of other quelites, but the inflorescences of *C. berlandieri* showed higher TPC values than several other *Amaranthus* spp. flowers [23] and values similar to those reported for flowers of common mullein (*Verbascum thapsus*) [24]. The highest values of phenolic compounds in boiled and oven-dried leaves are similar to the values reported by Godínez-Santillan et al. [25] in *C. aconitifolius* leaves previously dried at 40 °C and boiled for 10 min. Other studies, such as Fauziah et al. [26], also observed an increase in the amount of phenolic compounds and the antioxidant capacity of samples boiled for 5, 10 and 15 min. It has been observed that the thermal treatment of different plant foods causes an increase or decrease in the amount of phenolic compounds depending on the type of plant matter and the time invested in the process [27]. Coupled with this thermal effect, oven drying may cause a greater extraction of the polyphenolic compounds that are attached to the cell wall and subcellular compartments; compounds which likely contain more hydroxyl groups, increasing hydrogen donation [25].

Regarding antioxidant capacity, heat treatment did not improve radical scavenging, as analyzed by DPPH, FRAP, and ABTS assays. The freeze-dried raw vegetable samples presented greater radical scavenging activity, but a decrease in antioxidant capacity was observed after heat treatment, except in the FRAP assay. This may be due to an intermediate oxidation state of the polyphenolic compounds produced by the increasing number of reducing sugars or some products that may be generated by the Maillard reaction during the thermal processing of foods, resulting in a lower amount of polyphenols and an increase in antioxidant capacity [28]. Furthermore, it should be recognized that the vegetable matrix Is complex and contains different bioactive compounds that may respond with greater antioxidant capacity. In the antioxidant capacity tests, the results may also be influenced by different reaction mechanisms with the antioxidant compounds of the plant samples [23,29]. This phenomenon has been described for other quelites such as *Portulaca oleracea*, *Amaranthus hypochondriacus*, and *Amaranthus caudatus* [10,23,30]. However, heat treatment is essential for inactivating enzymes such as polyphenol oxidase and several peroxidases [27].

**Figure 1:**
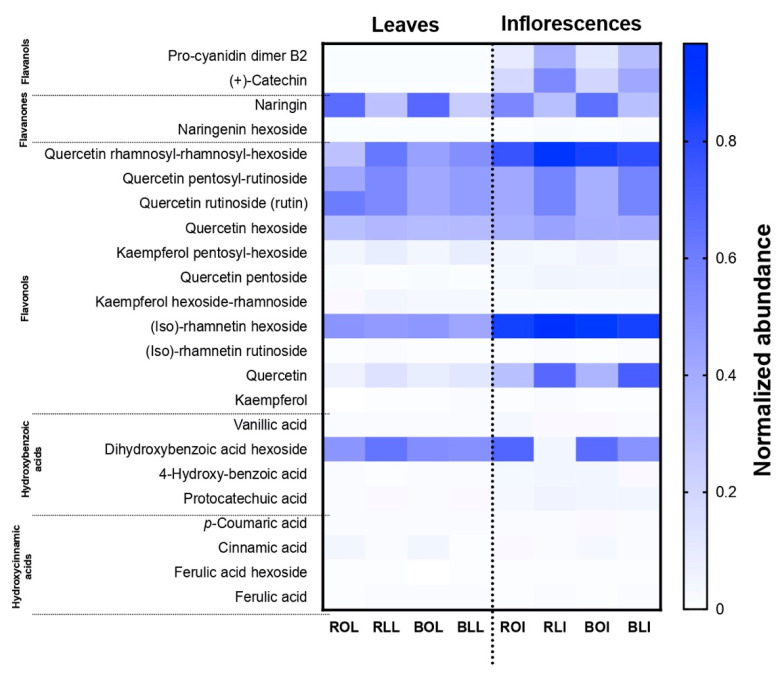
Normalized abundance of individual phenolic compounds of raw and boiled *C. berlandieri* leaves and inflorescences after processing by oven drying and lyophilization.

Individual phenolic compounds (Table 3) were identified by their exact molecular mass, by which 23 compounds were found (Table S1). Figure 1 shows the normalized abundance of each compound in the *C. berlandieri* samples, based on the amounts displayed in Table 3. Flavanols, flavanones, and flavonols were more abundant in the inflorescences than in the leaves, particularly quercetin-rhamnosyl-rhamnosyl-hexoside, (iso)-rhamnetin hexoside, and quercetin, coinciding with the higher amount of TF in inflorescences than in leaves, presented in Table 3. However, naringin, quercetin-rhamnosyl-rhamnosyl-hexoside, quercetin rutinoside, (iso)-rhamnetin hexoside, and dihydroxybenzoic acid hexoside were more abundant in the leaves. Quercetin rutinoside has been reported as one of the main flavanols of *C. berlandieri* and *C. ambrosioides* species [10,31], whereas kaempferol has not been previously reported for *C. berlandieri.* Procyanidin B2 was found, but only in the inflorescences. There are some current studies of the genus *Chenopodium* spp. that show it has hepatoprotective, antioxidant, antitumor, antibacterial, chemopreventive, and anticancer biological properties [2], probably due to its bioactive compounds.

The samples of raw freeze-dried leaf and inflorescences (RLL and RLI) presented higher amounts of individual phenols than the raw oven-dried raw samples of either leaves or inflorescence (ROL and ROI). Similarly, as mentioned above, after boiling, the oven-dried leaves and oven-dried inflorescence samples showed an increase in the quantity of phenolic compounds, while the lyophilized leaves and lyophilized inflorescences showed a decrease.

The results are the average normalized abundance of three independent experiments in triplicate. A min–max normalization was conducted as: (sample–min)/(max–min). BLI: Boiled lyophilized inflorescences; RLI: Raw lyophilized inflorescences; BOI: Boiled oven-dried inflorescences; ROI: Raw oven-dried inflorescences; BLL: Boiled lyophilized leaves; RLL: Raw lyophilized leaves; BOL: Boiled oven-dried leaves; ROL: Raw oven-dried leaves.

Coinciding with the results presented in Table 2. (+)-Catechin, kaempferol, and ferulic acid were found in the RLL but not in the ROL. However, after boiling the boiled oven-dried leaves (BOL), these compounds appeared. This may be due to a release of low-molecular-weight phenolic compounds that are bound to the plant fiber and were probably not released during oven drying [32]. In general, boiling favored the increase in phenolic compounds in the oven-dried samples and caused a slight decrease in the amount of some compounds in the freeze-dried samples, as shown in Table 3.

### 2.3. Principal Component Analysis (PCA) of Oven-Drying and Lyophilization Clustering

**Figure 2:**
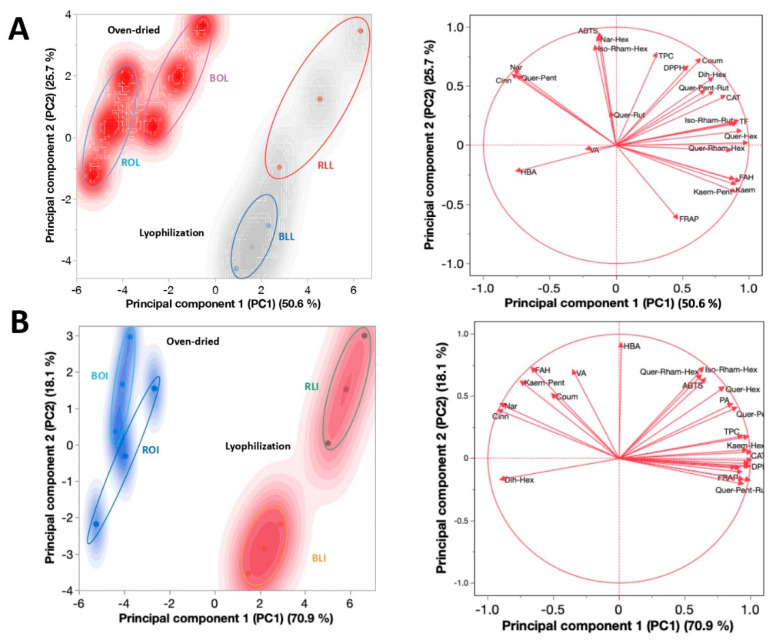
Principal component analysis (PCA) of *C. berlandieri* (**A**) leaves and (**B**) inflorescences, clustered by drying process. BLI: Boiled lyophilized inflorescences; RLI: Raw lyophilized inflorescences; BOI: Boiled oven-dried inflorescences; ROI: Raw oven-dried inflorescences; BLL: Boiled lyophilized leaves; RLL: Raw lyophilized leaves; BOL: Boiled oven-dried leaves; ROL: Raw oven-dried leaves. Blue and red areas indicate dots areas given by the cluster analysis.

Figure 2 shows the PCA analysis of the variables and phytochemicals evaluated in raw and boiled *C. berlandieri* leaves (Figure 2A) and inflorescences (Figure 2B), clustered by drying process.

Two components explained >80% of the variance (Table 4) in both the leaves and inflorescences. Moreover, a differential clustering was observed for the leaves and inflorescences depending on the drying process: ABTS and phenolics such as naringin, cinnamic acid, quercetin pentoxide, and ferulic acid hexoside were the components most affected by oven drying. In contrast, TPC, DPPH, *p*-coumaric acid, dihydroxybenzoic acid hexoside, quercetin-hexoside, and protocatechuic acid were the most affected by lyophilization. Based on the loading of each variable on each component (Table S2), it was shown that quercetin hexoside, naringenin hexoside, TPC, and DPPH were the most influential variables in principal component 1 (PC1). In contrast, ABTS, naringenin hexoside, vanillic acid, (Iso)-rhamnetin-hexoside, and ferulic acid hexoside were the most influential variables in PC2. The close relationship between the drying process and polyphenolic composition is explained by variations in the abundance of phenolics from dried plant material, which is critical considering that quelites are sold in both fresh and dried forms [5]. During food drying, the generation and accumulation of different bioactive compounds that could have antagonistic or synergistic effects upon each other, or upon other constituents of the sample occurs; these chemical interactions are still under investigation [33].

The analysis of the phenolic compound content and the antioxidant capacity of both botanical structures (leaves and inflorescences) suggests that it may be useful to include inflorescence in the human diet as a food or ingredient in different food dishes. In fact, the inflorescence of some quelites is the main edible product, such is the case of *Chenopodium berlandieri* ssp. *nuttaliiae* (huauzontle), which has been rarely studied.

## 3. Materials and Methods

### 3.1. Chemical Reagents

Ethanol, absolute methanol, sodium carbonate, aluminum trichloride, sodium hydroxide, sodium nitrate, potassium persulfate, hydrochloric acid, sodium acetate, glacial acetic acid, ferric chloride hexahydrate, gallic acid, catechin, 6-hydroxy-2,5,7,8-tetramethylchroman-2-carboxylic acid (Trolox), 2,2-diphenyl-1-picrylhydrazyl radical (DPPH), 2,4,6-tris (2-pyridyl)-s-triazine (TPTZ), 2,2′-azino-bis(3-ethylbenzothiazoline-6-sulfonic acid) (ABTS), Folin–Ciocalteu reagent, and phenolic HPLC-grade standards (*p*-coumaric acid, hydroxybenzoic acid, hesperidin, quercetin rutinoside, and rutin) were purchased from Sigma-Aldrich (St. Louis, MO, USA).

### 3.2. Plant Material and Study Design

Different samples of *Chenopodium berlandieri* were collected during August–September 2021 in La Barreta (Queretaro, Mexico), a town located at Lat. 20°49′47.6″ N and Lon. 100°30′11.9″ West and 2150 m above sea level. Plants were identified and registered (code: 00006282) by a specialist from the Jerzy Rzedowski Herbarium of the Universidad Autonoma de Queretaro. The study used a 2^3^ factorial design according to the following variables: (1) the botanical part analyzed: leaves or inflorescences; (2) the type of drying: oven-drying or lyophilization; (3) whether the samples were left raw or were boiled before the corresponding analyses. This resulted in 8 groups of samples: boiled lyophilized inflorescences (BLI); raw lyophilized inflorescences (RLI); boiled oven-dried inflorescences (BOI); raw oven-dried inflorescences (ROI); boiled lyophilized leaves (BLL); raw lyophilized leaves (RLL); boiled oven-dried leaves (BOL); and raw oven-dried leaves (ROL).

### 3.3. Drying Process

Immediately after collection, the samples were manually cleaned, separating the leaves and inflorescences. The samples were divided into two equal parts to be dried in an oven or by lyophilization. For the oven-drying procedure, the samples (leaves and inflorescences) were placed in a forced-ventilation oven (FX 1375, Shel Lab, Cornelius, OR, USA) at 40 °C for 48 h. For the lyophilization procedure, the samples were frozen at −80 °C for 24 h, and then placed in a freeze dryer (Scientz-10N, Ningbo Scientz Biotechnology Co., Zhejiang, China) at −60 °C and 1 Pa. Once dried, samples were ground in an electric blender (Thomas Model 4 Wiley Mill^®^, Thomas Scientific, Swedesboro, NJ, USA) and sieved through a 500 µm mesh. The resulting powders were then placed in sealed bags and stored at −80 °C for further analysis.

### 3.4. Proximate Chemical Analysis

For the raw leaves and inflorescences, analysis was performed according to AOAC (Association of Analytical Communities) procedures [34], as follows: quantification of ash (method 942.05), protein (method 920.87), lipids (920.39), moisture (method 925.10), lipids (method 920.39), and dietary fiber (method 962.09). Total carbohydrates were calculated by difference, according to equation [12]. Determinations were performed in triplicate in each analysis and the results were expressed as a percentage by weight of dry matter.

Total carbohydrates (%) = 100 − % (moisture + fiber + fat + ash + protein)(1)

### 3.5. Extraction, Identification, and Quantification of Phenolic Compounds

#### 3.5.1. Methanolic Extraction of Raw and Boiled Samples

**Figure 3:**
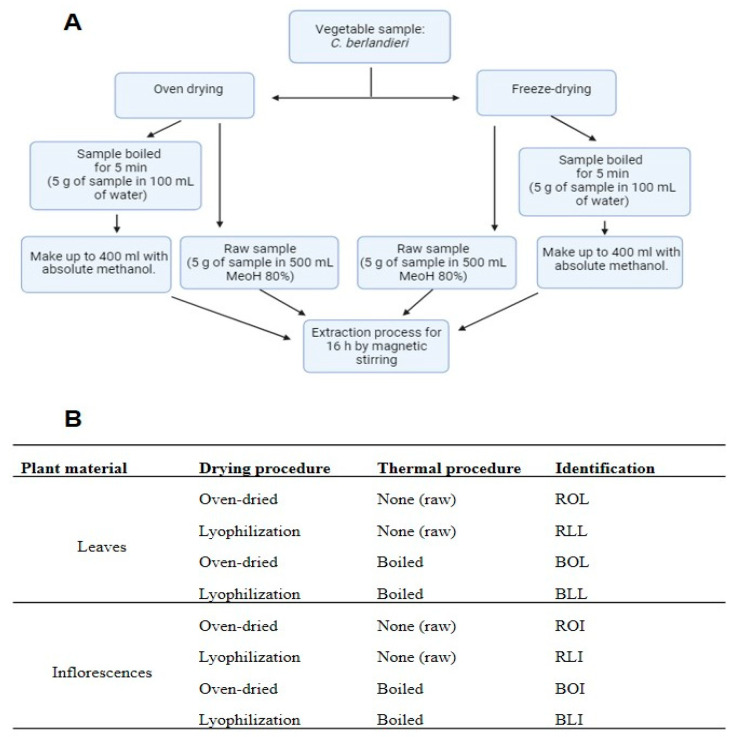
Methodology of the drying and extraction process of *C. berlandieri* (**A**). Identification of the samples used in this study (**B**).

The dried samples (oven dried and freeze dried) were separated into two groups, the first group was left as is (raw) and the second part was boiled, as follows. The sample was mixed with distilled water (5 g in 100 mL), heated at 100 °C for 5 min, and then cooled to room temperature. The amount lost from the initial 100 mL of distilled water was volumetrically completed. Subsequently, it was topped up to 400 mL with absolute methanol to obtain a proportion of 80:20 *v*/*v*. Subsequently, a hydroalcoholic extraction was carried out as described in Figure 3. The raw samples were mixed directly in a proportion of 5 g of plant sample to 500 mL of the hydroalcoholic dilution (80:20 *v*/*v*, again, absolute methanol to water). All extractions were left for 16 h under constant stirring (100 rpm) at room temperature (25 ± 1 °C) protected from light. Then extracts were filtered through Whatman No. 40 paper and rota-evaporated (R-200, Büchi, Essen, Germany) at 40 °C and 100 mm Hg pressure. The resulting solution was lyophilized as described above. The powdered extracts were packed in sealed bags, protected from light, and stored at −80 °C for further analysis.

#### 3.5.2. Spectrophotometric Determination of Total Phenolic Compounds (TPC) and Total Flavonoids (TF)

The total phenolic compounds (TPC) were quantified as reported by Singleton et al. [35] using the Folin–Ciocalteu reagent, and the results were expressed in mg gallic acid equivalents (GAE)/g sample. The total flavonoids (TF) were determined as described by Zhishen et al. [36], and the results were reported in mg of (+)-catechin equivalents/g sample.

#### 3.5.3. Identification and Quantification of Individual Phenolic Compounds by UPLC-DAD-ESI-QToF/MS

Individual phenolic compounds were identified and quantified by ultra-high performance liquid chromatography (UPLC) coupled to diode array detection (DAD) with electrospray ionization (ESI) coupled to a quadrupole time-of-flight (QToF) mass spectrometry (MS) [37]. Before the injection (2 µL sample) into the equipment, 25 mg of lyophilized extract was resuspended in 500 µL UPLC-grade water, and the sample was separated on a BEH Acquity C18 column (2.1 × 100 mm, 1.7 µm granule size) (Waters Corp., Milford, MA, USA) at 35 °C. The separation was performed using two solvents: MS-grade water adjusted with 0.1% formic acid (A) and 100% MS-grade acetonitrile (B) using the gradient conditions (0.5 mL/min) as follows: 0% B (0 min), 15% B (2.5 min), 21% B (10 min), 90% B (12 min), 95% B (13 min), and 0% B (15 min). The absorbances were read at 214, 280, 320, and 360 nm. For the quantification of individual compounds, commercial HPLC-grade standards of procyanidin dimer B2, (+)-catechin, naringin, rutin, quercetin, kaempferol, vanillic acid, 4-hydroxybenzoic acid, protocatechuic acid, *p*-coumaric acid, cinnamic acid, and ferulic acid were used. The mass spectrometer was operated under the following conditions: capillary voltage: 2.0 kV; cone voltage: 40 eV; low-collision energy: 6 V; high-collision energy: 15–45 V; source temperature: 120 °C; cone gas flow: 50 L/h, desolvation gas (N_2_) flow: 800 L/h (450 °C). Data acquisition was carried out in negative ionization mode (ESI^-^) with a mass range 100–1200 Da. A leucine enkephalin solution (50 pg/mL) was used to lock mass correction (10 µL/min). Compounds were identified through the exact mass analysis of pseudomolecular ions (mass error < 5 ppm), isotope distribution, and fragmentation pattern. The results were expressed as µg of each compound/g lyophilized extract.

#### 3.5.4. Antioxidant Capacity

Antioxidant capacity was determined by three different methods: the 1,1-diphenyl-2-picrylhydrazyl (DPPH) method reported by Brand-Williams et al. [38], the 2,2-azino-bis(3-ethylbenzothiazoline)-6-sulfonic acid (ABTS) method reported by Ozgen et al. [39], and the ferric reducing antioxidant power (FRAP) method reported by Benzie and Strain [40]. Results from the 3 methods were reported in µmol equivalents of Trolox/g sample. For all the assays, a Trolox standard curve was established (0–700 µM).

### 3.6. Statistical Analysis

The results were expressed as the mean ± SD of three independent experiments in triplicates. An analysis of variance (ANOVA) followed by post-hoc Tukey–Kramer’s test was conducted, establishing significance as *p* < 0.05. A Principal Component Analysis (PCA) was also carried out using a correlation matrix between the analyzed variables and the drying method (oven-dried or lyophilized), and all the analysis were performed in JMP v. 17 (SAS, Cary, NC, USA).

## 4. Conclusions

The study revealed that freeze-drying could better preserve the phenolic compounds and antioxidant capacity of *C. berlandieri* than oven drying. Despite the decrease in individual phenolic compounds in the freeze-dried samples after heat treatment, they present acceptable amounts of phenolic composition, mainly in the inflorescence. Several popular types of quelites are boiled before being consumed, which, as observed in the present study, results in some loss of phenolic compounds. Thus, plant products that are consumed dry and without any heat treatment may be an important source of phenolic compounds with potential antioxidant activity. In the present investigation, it was observed that the inflorescence is richer in phenolic compounds and antioxidant capacity than leaves. However, both plant samples show an interesting content of phenolic compounds that may have beneficial effects on health. Further in vitro and in vivo investigations should be performed to assess these biological effects.

## Figures and Tables

**Table 1:** Proximate chemical analysis of raw leaves and inflorescences of *C. berlandieri* by dry matter weight.

Component (%)	ROL	RLL	ROI	RLI
Moisture	2.91 ± 0.08 ^a^	2.66 ± 0.04 ^a^	2.59 ± 0.01 ^a^	2.80 ± 0.02 ^a^
Protein	23.17 ± 1.79 ^a^	23.97 ± 0.55 ^a^	23.28 ± 0.72 ^a^	23.00 ± 0.10 ^a^
Lipids	0.71 ± 0.02 ^b^	0.72 ± 0.02 ^b^	0.55 ± 0.54 ^c^	1.06 ± 0.05 ^a^
Ash	24.19 ± 0.22 ^a^	22.59 ± 0.04 ^a^	15.95 ± 0.18 ^b^	14.71 ± 0.33 ^b^
Total carbohydrates	29.79 ± 0.26 ^d^	32.45 ± 0.26 ^c^	41.71 ± 0.02 ^a^	35.67 ± 0.23 ^b^
Dietary fiber	19.23 ± 1.96 ^b^	17.61 ± 2.45 ^c^	15.92 ± 0.40 ^d^	22.76 ± 0.93 ^a^

The results are expressed in percentages as the mean ± SD of three independent experiments. Different letters by row express significant differences (*p* < 0.05) by Tukey–Kramer’s test. ROL: Raw oven-dried leaves; RLL: Raw lyophilized leaves; ROI: Raw oven-dried inflorescences; RLI: Raw lyophilized inflorescences.

**Table 2:** Effect of drying on the total phenolic composition and antioxidant capacity of raw and boiled leaves and inflorescences of *C. berlandieri* ssp. *berlandieri*.

Sample	TPC (mg GAE/g LE)	TFC (mg CE/g LE)	DPPH (µM TE/g LE)	ABTS (µM TE/g LE)	FRAP (µM TE/g LE)
ROL	23.29 ± 0.11 ^a^	6.79 ± 0.11 ^c^	100.32 ± 171 ^b^	384.15 ± 20.9 ^ab^	528.21 ± 15.9 ^b^
RLL	26.38 ± 0.08 ^a^	9.24 ± 0.08 ^a^	116.58 ± 0.93 ^a^	387.01 ± 28.3 ^ab^	545.49 ± 25.6 ^b^
BOL	26.09 ± 0.13 ^b^	8.02 ± 0.07 ^b^	114.68 ± 1.85 ^a^	424.80 ± 11.1 ^a^	525.12 ± 8.35 ^b^
BLL	22.06 ± 0.10 ^d^	7.92 ± 0.13 ^b^	101.52 ± 1.20 ^b^	341.82 ± 20.1 ^b^	635.00 ± 16.6 ^a^
ROI	27.69 ± 0.10 ^c^	11.99 ± 0.44 ^c^	117.76 ± 0.18 ^c^	554.52 ± 15.8 ^b^	656.60 ± 20.3 ^bc^
RLI	52.52 ± 0.15 ^a^	23.14 ± 0.13 ^a^	337.15 ± 7.75 ^a^	732.99 ± 61.3 ^a^	803.52 ± 53.3 ^a^
BOI	26.60 ± 0.11 ^d^	10.92 ± 0.07 ^d^	119.63 ± 0.21 ^c^	536.48 ± 36.3 ^b^	570.19 ± 20.6 ^c^
BLI	34.77 ± 0.18 ^b^	20.30 ± 0.10 ^b^	255.40 ± 3.39 ^b^	501.26 ± 38.9 ^b^	757.22 ± 52.8 ^ab^

The results are expressed as the mean ± SD of three independent experiments in triplicate. Different letters indicate significant differences (*p* < 0.05) by Tukey–Kramer’s test for the leaves or in-florescences. TPC: total phenols, TFC: total flavonoids, DPPH: 2,2-diphenyl-1-picrylhydrazyl, ABTS: 2,2-355 azino-bis(3-ethylbenzothiazoline-6-sulfonic acid), FRAP: ferric reducing antioxidant power, GAE: gallic acid equivalent, CE: catechin equivalent, LE: freeze dried extract, TE: Trolox equivalent, BLI: Boiled lyophilized inflorescences, RLI: raw lyophilized inflorescences, BOI: boiled oven-dried inflorescences, ROI: raw oven-dried inflorescences, BLL: boiled lyophilized leaves, RLL: raw lyophilized leaves, BOL: boiled oven-dried leaves, ROL: raw oven-dried leaves.

**Table 3:** Phenolic compounds from raw and boiled oven-dried and lyophilized *C. berlandieri* ssp. *berlandieri* leaves and inflorescences, quantified by UPLC-DAD-ESI-QTOF-MS (µg of each phenolic compound/g of lyophilized extract).

				Samples				
Compounds	ROL	RLL	BOL	BLL	ROI	RIL	BOI	BLI
*Flavanols*								
Pro-cyanidin dimer B2 *	n. d.	n. d.	n. d.	n. d.	599.3 ± 209.9 ^c^	2287.0 ± 33.4 ^a^	759.8 ± 148.5 ^c^	1939.5 ± 384.9 ^b^
(+)-Catechin *	n. d.	39.9 ± 0.6 ^d^	20.7 ± 10.0 ^d^	11.2 ± 1.4 ^d^	1151.4 ± 193.7 ^c^	3326.8 ± 41.9 ^a^	1204.0 ± 100.5 ^c^	2538.7 ± 238.2 ^b^
*Flavanones*								
Naringin *	4032.3 ± 474.0 ^a^	1768.5 ± 511.3 ^c^	4146.0 ± 215.9 ^a^	1480.6 ± 42.4 ^c^	3371.8 ± 170.9 ^b^	1863.5 ± 97.9 ^c^	3972.9 ± 86.4 ^a^	1868.7 ± 168.4 ^c^
Naringenin hexoside	45.8 ± 1.3 ^c^	42.9 ± 11.8 ^c^	46.0 ± 7.8 ^c^	31.6 ± 1.1 ^cd^	24.2 ± 7.8 ^d^	91.9 ± 9.3 ^a^	21.6 ± 1.8 ^d^	61.3 ± 1.0 ^b^
*Flavonols*								
Quercetin rhamnosyl-rhamnosyl-hexoside	1806.2 ± 122.3 ^g^	3766.8 ± 310.3 ^d^	2741.0 ± 52.7 ^fg^	3152.8 ± 64.3 ^e^	4654.4 ± 29.9 ^bc^	5570.0 ± 356.5 ^a^	5098.3 ± 35.2 ^ab^	4784.6 ± 6.8 ^b^
Quercetin pentosyl-rutinoside	2520.1 ± 536.9 ^bcd^	3350.6 ± 1162.1 ^abc^	2520.7 ± 636.9 ^bcd^	2791.4 ± 73.9 ^abcd^	2512.8 ± 408.2 ^cd^	3496.3 ± 21.7 ^a^	2341.5 ± 77.4 ^d^	3487.8 ± 5.3 ^a^
Quercetin rutinoside (rutin) *	3659.9 ± 5.8 ^d^	7652.8 ± 29.2 ^a^	4858.7 ± 905.0 ^c^	5695.7 ± 63.8 ^b^	3702.4 ± 358.2 ^d^	5454.6 ± 148.7 ^bc^	3998.5 ± 175.7 ^d^	5098.8 ± 70.8 ^b^
Quercetin hexoside	1827.9 ± 16.3 ^d^	2050.5 ± 29.1 ^c^	1966.5 ± 38.3 ^c^	1987.3 ± 46.3 ^c^	2334.2 ± 169.0 ^b^	2686.1 ± 146.2 ^a^	2363.2 ± 30.1 ^b^	2388.7 ± 63.3 ^b^
Kaempferol pentosyl-hexoside	255.5 ± 17.9 ^bd^	526.4 ± 51.6 ^a^	260.3 ± 0.7 ^bd^	537.50 ± 37.0 ^a^	241.7 ± 8.8 ^de^	201.8 ± 13.9 ^ce^	296.9 ± 9.4 ^b^	183.8 ± 11.5 ^c^
Quercetin pentoside	56.5 ± 5.2 ^d^	46.0 ± 0.3 ^d^	57.6 ± 0.9 ^d^	45.8 ± 1.7 ^d^	207.3 ± 22.3 ^c^	285.6 ± 6.7 ^a^	232.2 ± 3.8 ^b^	241.5 ± 14.3 ^b^
Kaempferol hexoside-rhamnoside	169.0 ± 36.1 ^bd^	246.2 ± 88.1 ^a^	175.8 ± 36.2 ^b^	193.7 ± 2.2 ^ab^	65.4 ± 12.3 ^c^	108.2 ± 0.8 ^cd^	74.7 ± 0.8 ^c^	95.8 ± 0.8 ^c^
(Iso)-rhamnetin hexoside	3052.9 ± 230.9 ^c^	2859.0 ± 453.6 ^cd^	2926.4 ± 140.9 ^cd^	2563.8 ± 177.3 ^d^	5114.9 ± 481.3 ^b^	5876.0 ± 197.8 ^a^	5292.4 ± 3.8 ^b^	5078.6 ± 140.1 ^b^
(Iso)-rhamnetin rutinoside	43.7 ± 3.8 ^bc^	59.8 ± 4.9 ^b^	42.5 ± 2.3 ^cd^	48.9 ± 1.2 ^bcd^	35.1 ± 9.0 ^d^	73.8 ± 2.8 ^a^	41.8 ± 0.5 ^cd^	56.8 ± 2.1 ^b^
Quercetin *	381.7 ± 151.6 ^c^	848.1 ± 213.9 ^c^	547.0 ± 264.0 ^c^	744.5 ± 163.8 ^c^	1872.1 ± 1235.0 ^b^	4099.0 ± 466.4 ^a^	2164.7 ± 336.0 ^b^	4393.1 ± 520.9 ^a^
Kaempferol *	n. d.	51.1 ± 15.0 ^b^	24.3 ± 0.2 ^c^	60.9 ± 3.8 ^b^	18.7 ± 0.5 ^c^	88.2 ± 7.9 ^a^	n. d.	53.1 ± 9.1 ^b^
*Hydroxybenzoic acids*								
Vanillic acid *	69.3 ± 20.6 ^e^	56.40 ± 0.4 ^e^	60.4 ± 3.7 ^e^	68.8 ± 2.4 ^e^	190.5 ± 27.6 ^a^	162.6 ± 21.4 ^c^	164.2 ± 9.8 ^bc^	124.9 ± 0.3 ^d^
Dihydroxybenzoic acid hexoside	2995.0 ± 618.9 ^b^	3842.30 ± 467.6 ^a^	3126.5 ± 214.8 ^b^	3136.9 ± 255.7 ^b^	4192.1 ± 448.0 ^a^	234.8 ± 10.3 ^c^	4073.9 ± 9.4 ^a^	3078.0 ± 21.2 ^b^
4-Hydroxy-benzoic acid *	67.1 ± 13.0 ^d^	n. d.	61.0 ± 2.5 ^d^	62.7 ± 1.3 ^d^	202.3 ± 29.2 ^bc^	234.0 ± 9.9 ^a^	234.5 ± 59.3 ^a^	161.9 ± 7.8 ^c^
3,4-Dihydroxy-benzoic acid (protocatechuic acid) *	80.6 ± 13.5 ^f^	134.30 ± 9.8 ^d^	107.1 ± 8.9 ^e^	148.0 ± 7.0 ^cd^	189.4 ± 30.6 ^c^	300.3 ± 13.9 ^a^	237.9 ± 23.3 ^b^	244.4 ± 5.6 ^b^
*Hydroxycinnamic acids*								
*p*-Coumaric acid *	63.9 ± 14.1 ^e^	84.30 ± 11.3 ^de^	73.2 ± 11.3 ^e^	64.3 ± 2.8 ^e^	106.3 ± 3.8 ^bc^	101.0 ± 8.7 ^cd^	126.0 ± 12.5 ^a^	106.3 ± 3.8 ^bc^
Cinnamic acid *	219.6 ± 37.2 ^a^	62.40 ± 1.5 ^c^	239.6 ± 24.9 ^a^	43.9 ± 2.8 ^c^	161.7 ± 18.9 ^b^	58.3 ± 0.3 ^c^	177.5 ± 11.7 ^b^	65.6 ± 10.9 ^c^
Ferulic acid hexoside	n. d.	27.80 ± 1.8 ^cd^	n. d.	21.1 ± 0.3 ^e^	38.4 ± 6.4 ^b^	31.1 ± 1.2 ^bc^	42.5 ± 1.4 ^a^	24.9 ± 1.0 ^de^
Ferulic acid *	n. d.	90.10 ± 5.7 ^a^	73.8 ± 3.6 ^a^	80.6 ± 4.5 ^a^	45.5 ± 12.5 ^b^	82.3 ± 5.7 ^a^	50.5 ± 9.3 ^b^	83.8 ± 9.6 ^a^

The results are the mean ± SD of three independent experiments in triplicate. Different letters in each row indicate significant differences by Tukey–Kramer’s test (*p* < 0.05). * Identification confirmed by comparison with commercial standards. BLI: Boiled lyophilized inflorescences; BLI: Boiled lyophilized inflorescences; BLL: Boiled lyophilized leaves; BOI: Boiled oven-dried inflorescences; BOL: Boiled oven-dried leaves; RLI: Raw lyophilized inflorescences; RLL: Raw lyophilized leaves; ROI: Raw oven-dried inflorescences; ROL: Raw oven-dried leaves; n. d.: not detected.

**Table 4:** Percentages and cumulative percentages of principal component analysis (PCA) analysis from *C. berlandieri* ssp. *berlandieri* (**A**) leaves and (**B**) inflorescences.

(**A**)	**Principal Component (PC)**	**Percentage**	**Cumulative Percentage**
	1	70.924	70.924
	2	18.074	88.998
	3	5.270	94.269
	4	3.158	97.426
	5	1.260	98.687
	6	0.879	99.566
	7	0.433	99.999
	8	0.001	100.000
(**B**)	1	69.529	69.529
	2	20.203	89.732
	3	4.919	94.651
	4	2.947	97.598
	5	1.176	98.774
	6	0.082	98.856
	7	0.404	99.260
	8	0.001	99.261

## Data Availability

Data will be available upon reasonable request.

## Supplementary Materials

The following supporting information can be downloaded at: https://www.mdpi.com/article/10.3390/molecules28207235/s1, Table S1: Phenolic compounds from *C. berlandieri* leaves and inflorescences, identified by UPLC-DAD-ESI-QToF/MS, Table S2: Loading of each variable on each component from the PCA analysis for *C. berlandieri* ssp. *berlandieri* (A) leaves and (B) inflorescences.
