# Supplementary material for: Impact of Drying Process on the Phenolic Profile and Antioxidant Capacity of Raw and Boiled Leaves and Inflorescences of Chenopodium berlandieri ssp. berlandieri
Source: Molecules. 2023 Oct 23;28(20):7235. doi: 10.3390/molecules28207235 (PMC10608939; doi:10.3390/molecules28207235)
Supplement: Supplementary file 1 [file molecules-28-07235-s001.zip › molecules-2617143-supplementary.pdf]

Supplementary Material

Impact of drying process on the phenolic profile and antioxidant capacity of raw and boiled leaves and inflorescences of *Chenopodium berlandieri* ssp. *berlandieri*

Supplementary Table S1. Phenolic compounds from *C. berlandieri* leaves and inflorescences, identified by UPLC-DAD-ESI-QToF/MS

N°	Identified compounds	RT (min)	Molecular formula	Expected mass (Da)	Observed mass (Da)	Mass error (ppm)	Fragments
Flavanols							
1	Pro-cyanidin dimer B2*	3.17	$C_{30}H_{26}O_{12}$	578.1424	578.1404	-3.5563	289.07178, 245.08154
2	(+)-Catechin*	3.39	$C_{15}H_{14}O_6$	290.0790	290.0784	-2.0368	245.08178, 109.02922
Flavanones							
3	Naringin*	4.87	$C_{27}H_{32}O_{14}$	580.1792	580.1803	1.9308	151.00383
4	Naringenin hexoside	7.28	$C_{21}H_{22}O_{10}$	434.1213	434.1217	0.8797	271.06143, 152.01138
Flavonols							
5	Quercetin rhamnosyl-rhamnosyl-hexoside	3.84	$C_{33}H_{40}O_{20}$	756.2113	756.2089	-3.1834	300.02825, 178.99880, 151.00365
6	Quercetin pentosyl-rutinoside	4.07	$C_{32}H_{38}O_{20}$	742.1956	742.1938	-2.5504	300.02847, 178.99886, 151.00379
7	Quercetin rutinoside (rutin)*	4.73	$C_{27}H_{30}O_{16}$	610.1534	610.1520	-2.3359	300.02884, 178.99918, 151.00408
8	Quercetin hexoside	5.03	$C_{21}H_{20}O_{12}$	464.0955	464.0967	2.7193	300.02896, 178.02768
9	Kaempferol pentosyl-hexoside	5.13	$C_{26}H_{28}O_{15}$	580.1428	580.1432	0.6679	284.03376
10	Quercetin pentoside	5.41	$C_{20}H_{18}O_{11}$	434.0849	434.0862	2.8964	300.02865, 179.03557, 150.03236
11	Kaempferol hexoside-rhamnoside	5.93	$C_{27}H_{30}O_{15}$	594.1585	594.1575	-1.6472	285.04136
12	(Iso)-rhamnetin hexoside	6.70	$C_{22}H_{22}O_{12}$	478.1111	478.1115	0.8259	314.04495, 151.00397
13	(Iso)-rhamnetin rutinoside	9.98	$C_{22}H_{22}O_{11}$	462.1162	462.1151	-2.4323	315.05057
14	Quercetin*	10.39	$C_{15}H_{10}O_7$	302.0427	302.0420	-2.1363	178.99844, 151.00334, 107.01371
15	Kaempferol*	11.28	$C_{15}H_{10}O_6$	286.0477	286.0484	2.1699	152.01126
Hydroxybenzoic acids							
16	Vanillic acid*	2.43	$C_8H_8O_4$	168.0423	168.0417	-3.5568	136.01586, 108.02128
17	Dihydroxybenzoic acid hexoside	2.77	$C_{13}H_{16}O_9$	316.0794	316.0807	3.9727	153.01841, 109.02883
18	4-Hydroxy-benzoic acid*	2.92	$C_7H_6O_3$	138.0317	138.0312	-3.7270	109.01557
19	3,4-Dihydroxy-benzoic acid (protocatechuic acid)*	4.10	$C_7H_6O_4$	154.0266	154.0265	-0.6641	153.01939, 137.02441, 109.02950
Hydroxycinnamic acids							
20	*p*-Coumaric acid*	3.08	$C_9H_8O_3$	164.0473	164.0468	-3.1319	119.05004
21	Cinnamic acid*	4.20	$C_9H_8O_2$	148.0524	148.0523	-0.8557	131.03771
22	Ferulic acid hexoside	4.80	$C_{16}H_{20}O_9$	356.1107	356.1112	1.2037	193.05123, 178.02775, 134.03753
23	Ferulic acid*	5.26	$C_{10}H_{10}O_4$	194.0579	194.0583	2.0288	178.02794, 134.03796

All ions were identified with the $[M-H]^-$ adduct. * These compounds were confirmed with commercial standards.

Supplementary Table S2. Loading of each variable on each component from the PCA analysis for *C. berlandieri* ssp. *berlandieri* **(A)** leaves and **(B)** inflorescences.

(**A**)

	**PC1**	**PC2**	**PC3**	**PC4**	**PC5**	**PC6**	**PC7**	**PC8**
TPC	0.082	0.290	-0.308	-0.003	0.105	-0.060	-0.038	-0.197
TF	0.247	0.078	-0.184	0.021	0.122	-0.041	0.010	-0.131
DPPH	0.145	0.251	-0.270	0.112	0.114	0.060	-0.060	0.900
ABTS	-0.033	0.355	-0.046	0.215	-0.074	-0.025	0.345	-0.010
FRAP	0.124	-0.234	0.289	0.236	-0.155	0.115	0.161	0.125
CAT	0.220	0.155	-0.191	-0.061	0.125	0.275	-0.116	-0.170
Nar	-0.204	0.238	0.026	0.129	-0.084	-0.003	-0.085	-0.037
Nar-Hex	-0.037	0.343	0.119	-0.094	-0.374	0.135	-0.113	-0.011
Quer-Rham-Hex	0.265	0.008	-0.080	0.084	-0.019	-0.069	-0.111	-0.080
Quer-Pent-Rut	0.179	0.173	0.302	-0.039	-0.230	-0.036	-0.331	0.028
Quer-Rut	-0.009	0.100	0.254	-0.545	0.184	0.569	0.080	0.062
Quer-Hex	0.253	0.046	-0.076	0.177	0.006	0.233	0.285	-0.096
Kaem-Pent	0.241	-0.147	0.109	-0.040	-0.006	-0.113	0.218	0.063
Quer-Pent	-0.198	0.220	0.053	0.225	0.194	0.005	0.109	-0.059
Kaem-Hex	0.196	0.169	0.248	-0.050	-0.251	-0.166	-0.346	0.021
Iso-Rham-Hex	-0.042	0.313	0.256	-0.023	-0.209	-0.237	0.419	0.069
Iso-Rham-Rut	0.242	0.069	0.103	-0.226	0.131	-0.244	0.039	0.003
Quer	0.237	0.071	0.193	0.091	-0.008	0.360	0.273	-0.016
Kaem	0.242	-0.125	0.040	0.177	-0.214	-0.008	-0.026	0.012
VA	-0.058	-0.013	0.424	0.230	0.606	-0.111	-0.167	0.031
Dih-Hex	0.196	0.215	0.162	0.036	0.232	-0.259	0.185	-0.047
HBA	-0.199	-0.083	0.209	0.359	-0.054	0.212	-0.124	0.057
PA	0.238	-0.108	0.098	0.236	0.037	0.146	-0.061	-0.027
Coum	0.171	0.274	0.118	0.036	0.189	0.044	-0.254	-0.117
Cinn	-0.209	0.225	-0.015	0.146	0.059	0.134	-0.103	-0.075
FAH	0.249	-0.114	0.024	-0.160	0.074	-0.137	0.022	0.020
FA	0.231	-0.017	-0.167	0.292	-0.071	0.173	-0.135	-0.130

(**B**)

	**PC1**	**PC2**	**PC3**	**PC4**	**PC5**	**PC6**	**PC7**	**PC8**
TPC	0.209	0.080	-0.230	-0.140	-0.002	-0.158	0.028	0.038
TF	0.221	-0.077	-0.022	-0.025	0.029	-0.062	-0.070	-0.016
DPPH	0.222	-0.027	-0.050	-0.120	0.022	-0.021	-0.052	-0.003
ABTS	0.146	0.281	-0.305	0.001	0.285	0.103	0.205	-0.272
FRAP	0.207	-0.048	-0.066	0.274	0.289	0.333	0.280	0.776
Cyanidin B2	0.219	-0.028	0.135	-0.028	0.051	-0.011	0.393	-0.194
CAT	0.224	-0.006	0.009	-0.054	0.016	-0.067	0.165	-0.077
Nar	-0.200	0.192	0.070	-0.020	-0.095	0.079	0.202	0.007
Nar-Hex	0.223	0.023	-0.056	-0.003	0.022	0.044	-0.175	-0.025
Quer-Rham-Hex	0.139	0.294	-0.058	-0.353	0.002	0.435	-0.375	0.049
Quer-Pent-Rut	0.209	-0.090	0.139	0.228	-0.092	-0.167	-0.193	0.046
Quer-Rut	0.210	-0.074	0.160	0.236	0.088	-0.045	-0.175	-0.080
Quer-Hex	0.178	0.252	-0.026	0.188	-0.087	0.257	-0.072	-0.152
Kaem-Pent	-0.167	0.273	0.142	-0.176	-0.083	0.177	0.173	0.028
Quer-Pent	0.199	0.180	0.104	-0.094	-0.229	-0.014	0.292	-0.014
Kaem-Hex	0.216	0.031	0.155	-0.037	-0.266	-0.135	-0.129	0.174
Iso-Rham-Hex	0.143	0.321	-0.044	0.141	-0.387	0.037	0.091	0.024
Iso-Rham-Rut	0.218	0.077	0.061	-0.054	-0.197	-0.042	-0.048	0.080
Quer	0.196	-0.034	0.345	0.243	-0.075	0.099	0.025	-0.134
Kaem	0.218	-0.017	-0.164	0.048	0.145	0.085	0.162	-0.239
VA	-0.077	0.309	-0.271	0.560	0.092	0.031	-0.294	-0.122
Dih-Hex	-0.200	-0.073	0.258	0.293	-0.074	0.108	-0.045	-0.035
HBA	0.004	0.406	0.085	-0.008	0.428	-0.610	-0.013	0.141
PA	0.191	0.193	0.197	-0.143	-0.042	-0.158	-0.170	0.173

Coum	-0.112	0.225	0.526	-0.141	0.395	0.223	-0.147	-0.052
Cinn	-0.204	0.171	0.059	0.123	-0.050	-0.062	0.242	-0.015
FAH	-0.148	0.319	0.018	0.140	-0.277	-0.130	0.085	0.140
FA	0.203	-0.032	0.314	0.138	0.135	0.030	0.171	-0.203

CAT: (+)-catechin; **Coum:** Coumaric acid; **Cinn:** Cinnamic acid; **Dih-Hex:** Dihydroxybenzoic acid hexoside; **FA:** Ferulic acid; **FAH:** Ferulic acid hexoside; **HBA:** 4-hydroxybenzoic acid; **Iso-Rham-Hex:** (Iso)-rhamnetin-hexoside; **Iso-Rham-Rut:** (Iso)-rhamnetin-rutinoside; **Kaem:** Kaempferol; **Kaem-Hex:** Kaempferol-hexoside; **Nar:** Naringin; **Nar-Hex:** Naringenin-hexoside; **PA:** 3,4-dihydroxybenzoic acid (protocatechuic acid); **PC:** Principal component; **Que:** Quercetin; **Quer-Hex:** Quercetin-hexoside; **Quer-Pent-Rut:** Quercetin-pentosyl-rutinoside; **Quer-Rham-Hex:** Quercetin-rhamnosyl-rhamnosyl-hexoside; **Quer-Rut:** Quercetin-rutinoside; **TF:** Total flavonoids; **TPC:** Total phenolic compounds; **VA:** Vanillic acid.
